# Progressive changes in composition of lymphocytes in lung tissues from patients with non-small-cell lung cancer

**DOI:** 10.18632/oncotarget.12264

**Published:** 2016-09-26

**Authors:** María del Mar Valenzuela-Membrives, Francisco Perea-García, Abel Sanchez-Palencia, Francisco Ruiz-Cabello, Mercedes Gómez-Morales, María Teresa Miranda-León, Inmaculada Galindo-Angel, María Esther Fárez-Vidal

**Affiliations:** ^1^ Department of Pneumology, San Cecilio University Hospital, Granada, Spain; ^2^ Department of Thoracic Surgery, Virgen de las Nieves University Hospital, Granada, Spain; ^3^ Institute for Biomedical Research, Virgen de las Nieves University Hospital, Granada, Spain; ^4^ Department of Pathology, School of Medicine, University of Granada, Granada, Spain; ^5^ Department of Statistics and Operative Research, School of Medicine, University of Granada, Granada, Spain; ^6^ Department of Biochemistry and Molecular Biology, School of Medicine, University of Granada, Granada, Spain

**Keywords:** immunological response, lung cancer, immunohistochemistry, flow cytometry, lymphocyte subsets

## Abstract

Immune cell infiltration is a common feature of many human solid tumors. Innate and adaptative immune systems contribute to tumor immunosurveillance. We investigated whether tumors evade immune surveillance by inducing states of tolerance and/or through the inability of some immune subpopulations to effectively penetrate tumor nests. Immunohistochemistry and flow cytometry analysis were used to study the composition and distribution of immune subpopulations in samples of peripheral blood, tumor tissue (TT), adjacent tumor tissue (ATT), distant non-tumor tissue (DNTT), cancer nests, cancer stroma, and invasive margin in 61 non-small-cell lung cancer (NSCLC) patients. A significantly higher percentage of T and B cells and significantly lower percentage of NK cells were detected in TT than in DNTT. Memory T cells (CD4^+^CD45RO^+^, CD8^+^CD45RO^+^) and activated T cells (CD8^+^DR^+^) were more prevalent in TT. Alongside this immune activation, the percentage of T cells with immunosuppressive activity was higher in TT than in DNTT. B- cells were practically non-existent in tumor nests and were preferentially located in the invasive margin. The dominant NK cell phenotype in peripheral blood and DNTT was the cytotoxic phenotype (CD56^+^ CD16^+^), while the presence of these cells was significantly decreased in ATT and further decreased in TT. Finally, the immunologic response differed between adenocarcinoma and squamous cell carcinoma and according to the tumor differentiation grade. These findings on the infiltration of innate and adaptative immune cells into tumors contribute to a more complete picture of the immune reaction in NSCLC.

## INTRODUCTION

Lung cancer accounts for the largest number of cancer-related deaths worldwide, and more than 85% of cases are non-small-cell lung cancer (NSCLC). There have been limited improvements in NSCLC therapy over the past few decades, and a five-year survival rate of only 16% has been estimated for NSCLC patients [1].

Immune cell infiltration is a common feature of many human solid tumors. Any type of infiltrating immune cell may be found in different tumors and in distinct localizations within tumors, i.e., in their core (center), invasive margin, or surrounds or in adjacent tertiary lymphoid structures [2]. The localization, density, and/or functional orientation of different immune cell populations can be beneficial or deleterious for patients. T lymphocytes play a vital role in the immune response against tumor development, and clinical and histopathological studies [3, 4] have described T-cell immune infiltrate as the most important predictor of patient survival [5–9]. However, cells infiltrating tumors are highly heterogeneous, and most components of myeloid and lymphoid compartments are represented. Furthermore, infiltrating immune cells can become activated through a perturbed phenotype and/or a functional profile that creates an environment conducive to T-cell suppression [10]. Immunosuppression leads to Th1/Th2 imbalance and Th2 drift, affecting anti-tumor immunity. Regulatory T cells (Tregs) are a population of T cells that suppress the activation of the immune system and maintain immune tolerance to self-antigens. Tregs can contribute to immunosuppressive or antitumor activity, allowing tumors to evade the immune barrier in epithelial malignancies [11]. Tregs are upregulated or activated in the tumor microenvironment, and a higher number of Tregs has been correlated with a worse prognosis in epithelial cancers, including lung cancer [12, 13]. Potential mechanisms underlying the immunosuppressive effects of Tregs may include the production of inhibitory cytokines, such as TGF-β and IL-10, and the suppression of T cell function by competitive binding of interleukin-2 (IL-2) *via* cell surface receptor CD25 (IL-2 receptor). In addition, several co-inhibitory molecules, such as cytotoxic T-lymphocyte-associated antigen 4 (CTLA-4) and glucocorticoid-induced tumor necrosis factor receptor (GITR), bind to ligands on effector T cells and directly contribute to the inhibitory function of Tregs [14].

There is a need for a more complete understanding of anti-tumor immune responses and of the role of NK cells in this process [15–17]. NK cells are innate lymphocytes with a natural ability to recognize and kill aberrant cells, including cancer cells [18–20]. There is increasing evidence that tumor-infiltrating NK cells have severe defects in their cell receptor repertoire, suggesting a local tumor-induced impairment of NK-cell function. Hence, the quality rather than quantity of intratumoral NK cells may account for their dysfunction. Intratumoral NK cells were found to express markedly lower levels of killer-cell immunoglobulin-like receptor (KIR) in comparison to peripheral blood NK cells from the same patients [21, 22]. Tumor-infiltrating NK cells without KIR expression, as non-educated cells, have no cytotoxic capacity [23, 24]. Recent studies also indicated that the phenotype of tumor-infiltrating NK cells without KIR expression was characteristic of immature and nonfunctional NK cells [25]. In support of this hypothesis, several studies showed that the NK-cell developmental program is not entirely fixed and that mature NK cells can be re-educated by their environment [26–28]. Hence, the tumor microenvironment may have a negative impact on NK-cell maturation.

Despite the importance of T cells and NK cells in tumors and tumor microenvironments, a comprehensive analysis of these lymphocytic cell populations has not been reported in NSCLC patients. All subsets of T cells and NK cells are present at the core and invasive margin of NSCLC tumors. Distinct functional populations of immune cells are found at different tumor localizations and their distribution pattern varies among cancer types, suggesting that different immune cell populations may have distinct roles in tumor control.

The objective of the present study was to analyze the composition and distribution of immune subpopulations in samples of peripheral blood, tumor tissue (TT), adjacent tumor tissue (ATT), distant non-tumor tissue (DNTT), cancer nests, cancer stroma, and invasive margin in NSCLC patients. The aim was to provide new insights into the distribution and phenotypic characteristics of different immune lymphocyte subpopulations in this disease.

## RESULTS

### Analysis of lymphocyte subsets in peripheral blood samples

Significant differences in NK cell, B cell, and T cell subsets were found between peripheral blood samples from NSCLC patients and healthy controls. In comparison to the controls, the patient peripheral blood samples had a significantly higher percentage (30.9 vs. 18.2 respectively; *p* < 0.001) and absolute number (887.2 vs. 465.7 cells/μl; *p* < 0.009) of NK cells and a significantly lower percentage (4.2 and 8.3, respectively; *p* < 0.001) and absolute number (128.3 vs. 196.8; *p* < 0.02) of CD20^+^ B cells. Significant differences between patients and controls were observed in the percentage and absolute number of CD4^+^ T cells but not in the absolute number of CD8^+^ T cells (p=0.634). Peripheral blood samples from patients showed a higher percentage of the following lymphocyte subsets in comparison to controls: CD4^+^ CD45RO^+^ 72.7 vs. 63.1 (*p* < 0.006), CD8^+^ CD45RO^+^ 41.64 vs. 33.90 (*p* < 0.02), CD4^+^ DR^+^ 7.7 vs. 3.9 (*p* < 0.001), CD8^+^ DR^+^ 9.9 vs. 6.3 (*p* < 0.001) and CD4^+^ T regulatory (Tregs) cells (CD127^low^ CD25^bright^) 6.9 vs. 5.9 (*p* < 0.02). Interestingly, a positive correlation was observed between the percentages of CD4^+^DR^+^, CD8^+^DR^+^, CD8^+^ CD45RO^+^, CD4^+^ CD39^+^, CD8^+^ CD39^+^ and CD4^+^ Treg subsets in the tumor sample and the percentages observed in peripheral blood. Thus, an increase in the percentage of CD4^+^DR^+^ cells in TT was accompanied by an increase in the percentage of CD4^+^DR^+^cells in peripheral blood (see below).

### Analysis of lymphocyte subsets in NSCLC patients

**Figure 1:**
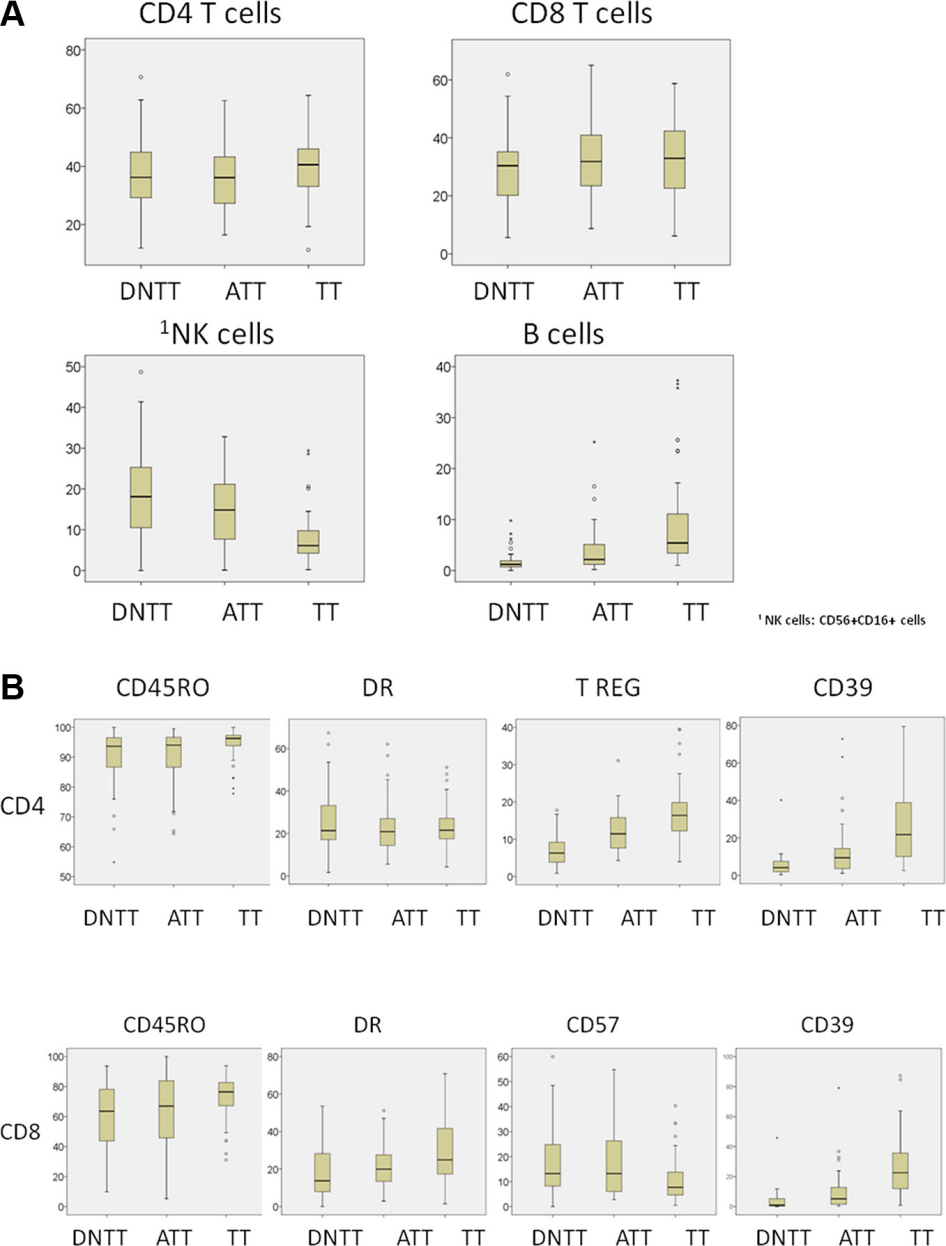
Analysis of immune cell density in tumor (TT), adjacent tumor (ATT), and distant non-tumor tissue (DNTT) samples from each patient Upper horizontal line of box plot, 75th percentile; lower horizontal line of box, 25th percentile; horizontal bar within box, median value (in %) of each subpopulation analyzed; upper horizontal bar outside box, 90th percentile; lower horizontal bar outside box, 10th percentile. Circles represent outliers.

Among this series of 61 patients operated for NSCLC, statistically significant differences in immune cell density were found among the distinct regions sampled in each patient (TT, ATT, and DNTT). Figure 1 summarizes the results for each subpopulation analyzed.

Percentages of CD3^+^ T cells and CD20^+^ B cells were higher in TT (79.7 and 10.7, respectively) than in ATT (77.4 and 5.22) and DNTT (74.27 and 2.82). The difference between TT and DNTT was significant for both subsets (*p* < 0.001).

Most of both CD4^+^ and CD8^+^ T cells had a memory-effector phenotype. However significant differences were observed in the percentage of these cells among the different regions. Memory T cells (CD4^+^ 45RO^+^ CD8^+^ CD45RO^+^) and Tregs cells (CD4^+^ CD25^bright^CD127^dim^) were more abundant in TT than in ATT or DNTT. CD4^+^ CD45RO^+^ count in TT (94.05) significantly differed (*p* < 0.008) from that in ATT (89.4) and DNTT (89.1). CD8^+^ CD45RO^+^ count in TT (73.5) significantly differed (*p* < 0.002) from that in ATT (64.9) and DNTT (61.8) samples. From a functional viewpoint, T- cells predominantly expressed Th1 and Th17 markers in TT. Percentages of granulocytes and Th17 cells were positively correlated in TT (*p* < 0.05).

The percentage of CD4^+^DR^+^ cells was elevated in TT but did not significantly differ from that observed in DNTT. The percentage of CD8^+^DR^+^ cells was significantly (*p* < 0.001) higher in TT (30.9) than in ATT (22.10) or DNTT (18.58). In TT, the percentages of CD4^+^DR^+^ cells and CD8^+^DR^+^ cells were inversely correlated with the percentage of CD20^+^ B cells (*p* < 0.01).

The percentage of Tregs cells was significantly higher (*p* < 0.001) in TT (17.5) than in ATT (12.5 or DNTT (7.1). The percentage of CD4^+^CD39^+^ was significantly (*p* < 0.001) higher in TT (25.4) than in ATT or DNTT, and the percentage of CD8^+^CD39^+^ cells was also significantly (*p* < 0.02) higher in TT (25.7) than in ATT or DNTT. Finally, an elevated percentage of fully-differentiated CD8^+^CD57^+^ cells was observed in the CD8^+^ T compartment (CD57 is a marker of T-cell exhaustion). In contrast to the above finding for CD39^+^, the percentage of CD8^+^ CD57^+^ T cells was significantly (*p* < 0.01) higher in DNTT (17.24) than in TT (11.2).

The balance between CD8/Tregs and Th1/Tregs has been shown to be important in tumor progression and prognosis in some studies of human cancer. The CD8/Tregs cell ratio was significantly (*p* < 0.001) higher in DNTT (17.8) than in TT (7.9), probably due to differences in T-regs and memory T- cells between the tissues. The Th1/T reg cell ratio was higher in DNTT (8.12) than in ATT (4.74) or TT (3.36), and the difference between TT and DNTT was significant (*p* < 0.006).

NK cells are characterized by the expression of CD56^+^ CD16^+^ and the absence of CD3. NK cells were increased in the peripheral blood and lung tissues of patients, with the highest percentage being observed in DNTT. The percentage was significantly (*p* < 0.001) lower in TT (7.39) than in ATT (14.48) or DNTT (19.74).

Various NK phenotypes were identified in the analyzed tissues depending on the CD56 and CD16 expression. The percentage of cytotoxic NK cells (CD56^+^ CD16^+^) was significantly (*p* < 0.001) lower in TT (22.8) than in ATT (40.29) or DNTT (44.45). In contrast, the percentage of CD56^bright^ CD16^−^ NK cells, (non-cytotoxic producing cytokines phenotype), was significantly higher TT (9.3) than in ATT (4.95; *p* < 0.03) or DNTT (2.00; *p* < 0.001). Finally, the percentage of double-negative CD56^−^ CD16^−^ or single-positive CD56^−^ CD16^+^ immature NK phenotypes was also higher in TT but statistical significance was not reached, likely attributable to the low number of samples.

### Analysis of TILS in relation to clinical-pathological features

Significantly higher percentages of CD4^+^CD39^+^ (*p* < 0.03) and CD8^+^CD39^+^ (*p* < 0.02) cells were found in samples from tumors with positron emission tomography (PET) standardized uptake value (SUV) ≤ 4.5 than in those from tumors with PET SUV > 4.5.

The percentage of CD4^+^CD39^+^ cells was significantly higher (*p* < 0.01) in samples from tumors less than two centimeters in size than in those from larger tumors.

The most common histological tumor type was squamous cell carcinoma (31 cases), followed by adenocarcinoma (23 cases). A significantly higher percentage of CD3^+^ T (*p* < 0.04), CD8^+^ (*p* < 0.03), CD8^+^ CD45RO^+^ (*p* < 0.01), CD4^+^DR^+^ (*p* < 0.02, CD8^+^DR^+^ (*p* < 0.05), and CD8^+^ CD39^+^ (*p* < 0.02) cells were found in TT from an adenocarcinoma than in TT from a squamous cell carcinoma.

### Immunohistochemical location and prognostic value of lymphocytic cell subpopulations

**Figure 2:**
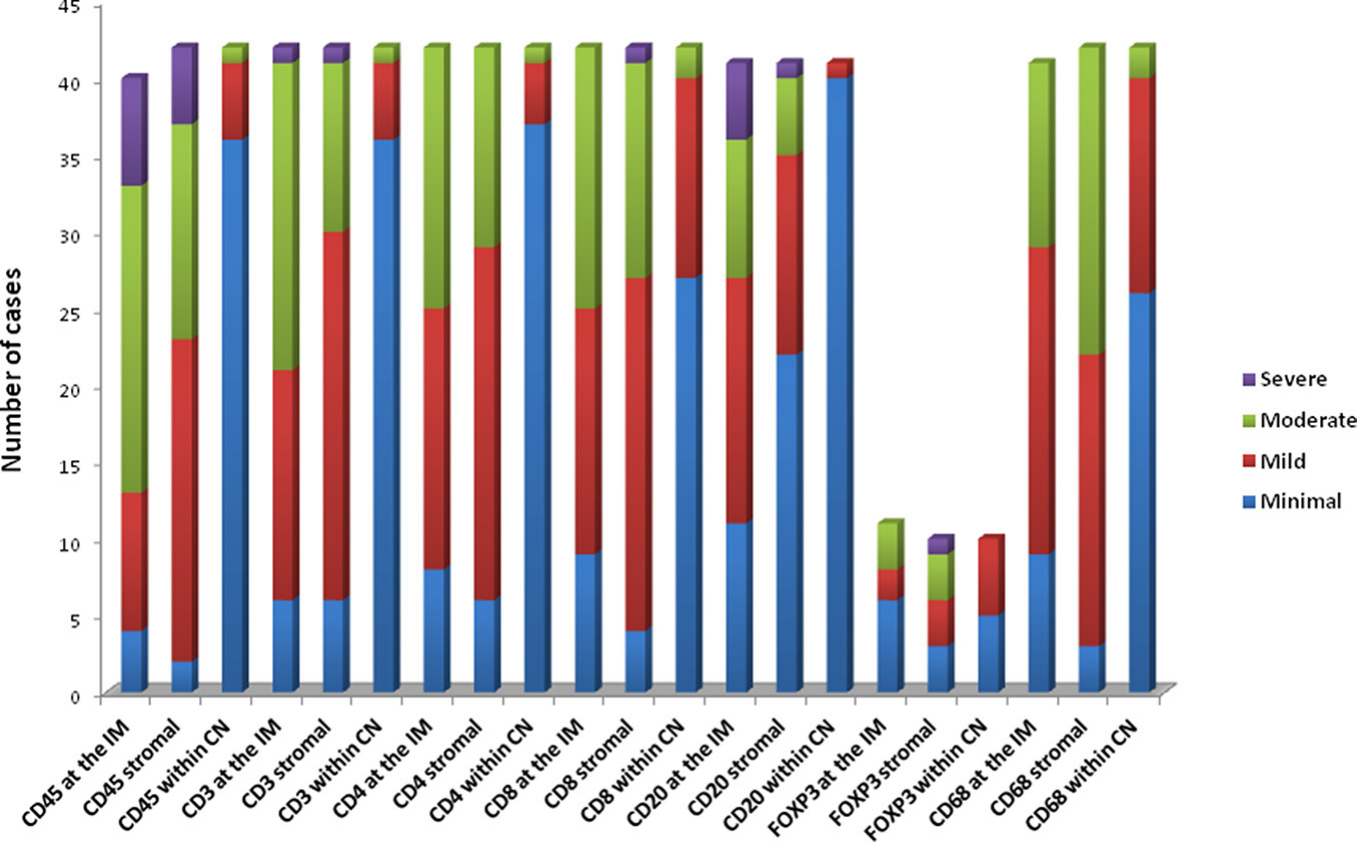
Immunohistochemistry results (A) Intensity of the immunohistochemical staining of T lymphocytes (including memory T cells), B-lymphocytes and CD 68 (macrophages) in the invasive margin (IM), stromal and within cancer nests (CN) for samples analyzed. Severe (score of 4), Moderate (score of 3), Mild (score of 2), Minimal (score of 1).

**Figure 3:**
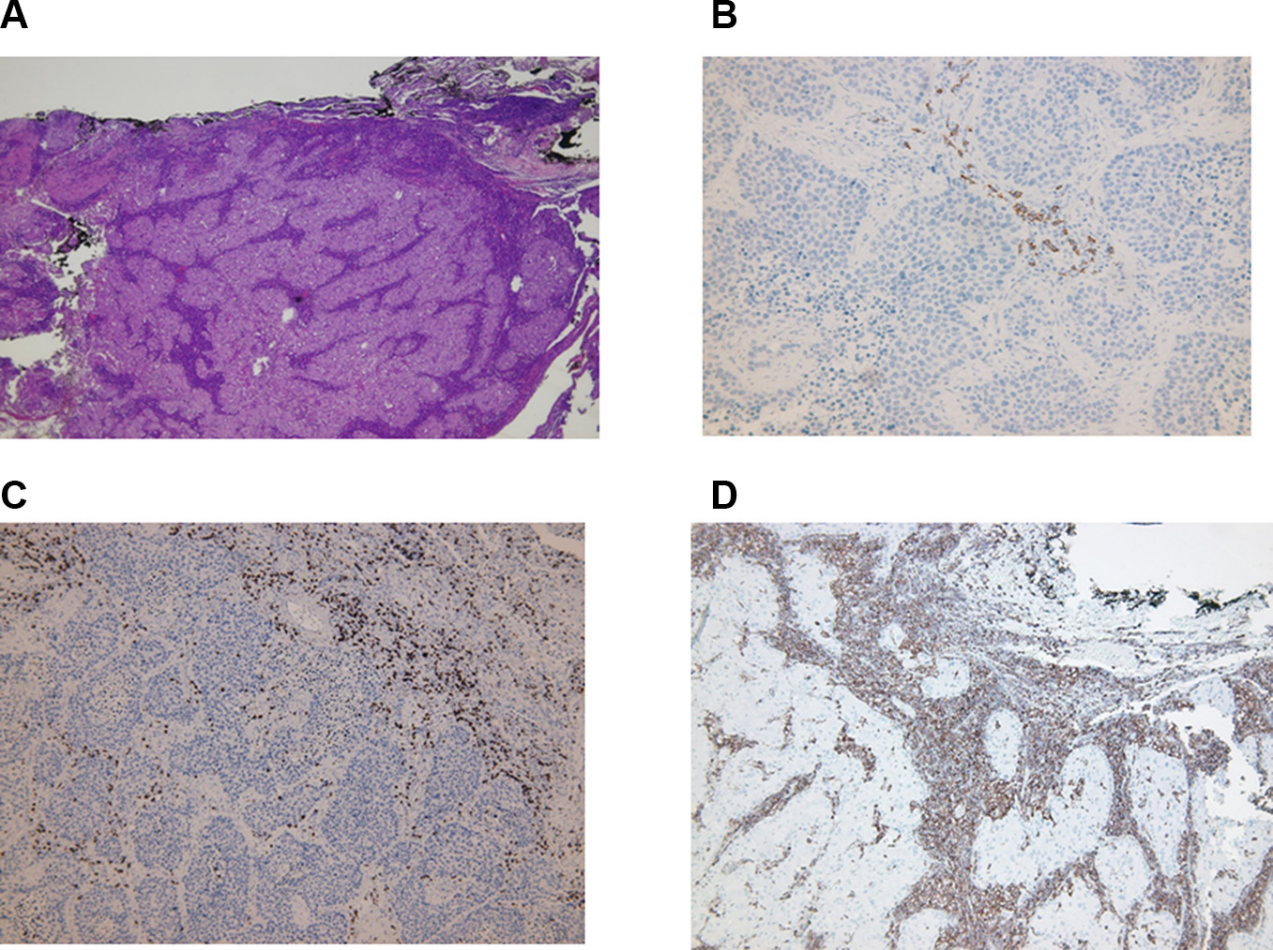
Immunohistochemistry results The infiltration of lymphocytes was more intense in invasive margin and stroma surrounding tumor nests. (**A**) Hematoxylin-eosin staining of tissue; magnification X4 followed by computer magnification. (**B**) B lymphocyte (CD20) staining in stroma; magnification X20 followed by computer magnification. (**C**) CD8^+^ staining in stroma and invasive margins; magnification X10 followed by computer magnification. (**D**) CD45^+^ staining in stroma and invasive margins; magnification X10 followed by computer magnification.

Location of lymphocytes and macrophages were studied in the invasive margin, stromal and within cancer nests in 42 patients (Figure 2A). The infiltration intensity of lymphocytes and macrophages was more evident in the invasive margin and in stroma surrounding tumor nests. Figure 3 provides an illustration of the immunohistochemistry results for the staining in stroma and invasive margins. In Figure 3A depicts an example of hematoxylin-eosin staining. Mildly intensive infiltration of cancer nests (score of 2) was observed in 15 of the 42 cases. CD4^+^ and CD8^+^ T lymphocytes were preferentially located in stroma and invasive margin (similar percentages of each), showing mild-moderate infiltration intensity. Figures 3C and 3D illustrate immunohistochemistry results for CD8 and CD45 staining, respectively.

There was a significantly higher frequency of CD8^+^ T- lymphocytes (15 cases) than of CD4^+^ T- lymphocytes (5 cases) in cancer nests.

The presence of Tregs in tissues was evaluated by analyzing the distribution and the absolute number of FoxP3^+^ Tregs. A higher number of Tregs were detected in stroma and tumor nest than in the corresponding invasive margin. There was a higher FoxP3^+^ cell count in stroma than in the other compartments. In 50% of studied cases, these cells were also observed within tumor nests, but with a mild infiltration intensity (score of 2). B cells were practically non-existent in tumor nests and were largely detected in the invasive margin and, to a lesser extent in the stroma. Figure 3B depicts an example of stromal staining for these cells. Finally, only a small number of cases showed the presence of macrophages (CD68) within cancer nests, with a score of 2; they were more prevalent in the stroma and even more so in the invasive margin in comparison to the other localizations.

Examination of the association of infiltration intensity and pattern with clinicopathological variables only revealed significant differences as a function of histological type and degree of differentiation. There was a higher T lymphocyte count in the stroma in adenocarcinomas than in squamous cell carcinomas (*p* < 0.03), attributable to differences in the CD4^+^ T subset (*p* < 0.02).

Significant differences as a function of the differentiation degree were only detected in T lymphocytes at the invasive margin, where CD4^+^ T cell infiltration was higher in well-moderately (69%) *versus* poorly (12.5%) differentiated tumors (*p* < 0.05), and CD8^+^ T cell infiltration was higher in well-moderately (64.3%) *versus* poorly (21.4%) differentiated tumors (*p* < 0.04). A higher percentage of CD8^+^ T cells was also found in well-moderately differentiated groups (*p* < 0.024) in tumor nests. A greater infiltration of FoxP3^+^ cells was observed in the invasive margin in poorly *versus* well-moderately differentiated tumors (*p* < 0.01). However, the percentages of these subsets in the cancer nests were higher in well-moderately *versus* poorly differentiated groups (*p* < 0.05). Finally, the percentage of CD68^+^ macrophages in the invasive margin was higher in well-differentiated tumor *versus* poorly differentiated tumors (70% vs. 23.5%, respectively).

## DISCUSSION

It is important to decipher the complex interactions of tumors with their microenvironments in order to understand anti-cancer defense systems [29]. The immune signature in NSCLC tumors was investigated in the present study, using a combination of immunohistochemistry and flow cytometry analysis. Comprehensive analysis of the majority of immune subsets that infiltrate NSCLC tumors demonstrated a wide host defense array and revealed the relevant role of different lymphocyte subsets in these patients. We analyzed the composition and distribution of immune subpopulations in peripheral blood, TT, ATT, DNTT, cancer nests, cancer stroma, and invasive margin, investigating the functional state of lymphocytes and the association of immune subsets with prognostic clinicopathological variables in NSCLC patients.

Our results showed a higher percentage of NK cells, memory T cells (CD4^+^CD45RO^+^, CD8^+^CD45RO^+^), activated T cells (CD4^+^DR^+^, CD8^+^DR^+^) and Tregs (CD4^+^CD25^bright^CD127^dim^) cells in the peripheral blood of NSCLC patients in comparison to the healthy controls, indicating a certain level of immune response in the patients. We cannot rule out the possibility that these differences were due to the presence of chronic obstructive pulmonary disease, the most frequent comorbidity among the patients. However, the changes in cell subsets observed may also be induced directly, at least to some extent, by the tumor cells and/or tumor microenvironment, as indicated by our analysis of the lymphocyte subsets in these patients. Thus, the percentages of activated T cells and CD4-Tregs in peripheral blood samples were positively correlated with those in tumor samples from these NSCLC patients. The subpopulation of CD4^+^ Tregs cells was also more abundant in patients than in controls, consistent with the immunosuppressive status of the patients, which was confirmed in the analyses of lung tissues. These data show that study of the proportion of T cell subsets in peripheral blood can provide valuable information. These observations confirm those of other researchers in lung cancer [30–33] although previous reports indicated that alterations in immunoregulatory T cells in lung cancer are more pronounced in bronchoalveolar lavage fluid obtained from both lungs than in peripheral blood [34].

Infiltration of the tumor by cytotoxic CD8^+^ T-cells and memory CD4^+^ CD45RO^+^ cells proved to have prognostic discriminatory power, leading to a novel scoring system strongly correlated with the clinical outcome [29]. Tumor-infiltrating CD8^+^ T lymphocytes play important roles in anti-tumor immune responses and have prognostic value in various cancer types. Thus, immune infiltration in both the center and invasive margin of human colorectal tumors was associated with a favorable clinical outcome, while a low density of T cells was associated with a poor prognosis [7, 10, 28, 29]. In most studies, immunoscores were based on immunohistochemistry techniques, which do not permit an in-depth characterization of the functional phenotype of T cells (activated or not, in an anergy state or not, or with maturation deficiencies or not). In the present study, an exhaustive and comprehensive analysis was carried using a combination of flow cytometry and immunohistochemistry, providing an in-depth characterization of the cellular components of a tumor biopsy specimen in comparison to DNTT and ATT samples from same patient. The results showed that T and NK cells are both highly heterogeneous in lung cancer and that some subpopulations may suppress the function of other immune cells, allowing the cancer cell to evade activation of immune system. The tumor samples showed a higher prevalence of cells with memory phenotype CD4^+^CD45RO^+^, a higher prevalence of CD4^+^ *versus* CD8^+^ T cells (CD4 > CD8), and a higher prevalence of Th1 *versus* Th17 phenotype, with a reduced presence of NK cells in TT. No significant differences in localization were found between CD4^+^ T cells and CD8^+^ T cells; however, CD8^+^ T and especially CD4^+^ T cells were excluded from direct contact with tumor and were trapped in the stroma or invasive margin (Figure 2). CD8^+^ T cells were observed in tumor nests in a few cases, but always at a low density (Figure 3). Stromal cells in the tumor microenvironment (macrophages and cancer-associated fibroblasts) prevent the access of lymphocytes to tumor nests, leading to their accumulation in the vicinity of tumors [35].

Nevertheless, our data suggest the presence of some immunosuppressive factors that act from the center of tumor towards the outside and may affect and significantly disrupt the function of some immune subsets. Percentages of T and B cells were higher in TT than in DNTT, at the expense of significant reduction in NK cells, and there was a higher percentage of memory T cells (CD4^+^CD45RO^+^, CD8^+^CD45RO^+^) and activated T cells (CD8^+^DR^+^) in TT. Parallel with this immune activation, TT showed: i) a higher percentage of Treg cells (CD4^+^ CD25^bright^CD127^dim^); ii) a lower CD8/Treg ratio, and iii) a lower Th1/Treg ratio and iv) higher percentage of T cells with immunosuppressive activity (CD4^+^CD39^+^ and CD8^+^CD39^+^) in comparison to DNTT (*p* < 0.001 and 0.02, respectively).

Tregs may suppress the function of immune cells and allow lung cancer cells to evade escape, likely *via* cell interaction-dependent or cytokine-mediated suppression. However, the balance between T-effector and T-regs in tumors is known to determine the functional outcome of immune responses [36], and both Th1/Treg and CD8/Treg ratios were lower in TT than in DNTT. Although the precise mechanism of Tregs-mediated suppression has yet to be fully elucidated [37–39], at least part of the immunosuppressive effect may be could be exerted by CD39. This ectoenzyme serves as an integral component of the suppressive machinery of Tregs, inactivating and converting extracellular ATP into adenosine and allowing the immune escape of tumors [40–42]. CD39, which mediates immunosuppressive functions, is also present on different immune cell subsets. In non-Tregs cells, the expression of CD39 on CD4 and CD8 cells has been associated with anergized [43] or exhausted T cells [44]. Furthermore, CD8^+^CD39^+^ T cells substantially inhibit IFNγ production by effector CD8^+^ T cells *via* the paracrine generation of adenosine [45]. A marked accumulation of CD39^+^ T cells (both CD4 and CD8) in the lung tumor tissue was observed in the present study.

The accumulation of CD8^+^CD39^+^ in TT may be the result of chronic and persistent stimulation and could therefore be generated within the tumor. Indeed, CD39 was found to be a specific pathological marker of exhausted CD8^+^ T cells in chronic viral infection in humans and mouse models [45, 46]. CD57 is also a marker for exhausted senescent-T cells [47], but the presence of CD39+ exhausted CD8^+^ T cells in TT contrasts with the absence of CD8^+^CD57^+^ cells in TT.

Previous studies found that an increase in CD8^+^CD57^+^ (senescent) cells is associated with malignancy. Fully-differentiated CD8^+^CD57^+^ cells were abundant in peripheral blood of both patients and controls who showed similar values, attributable to the age-matching of the groups, but no CD8^+^CD57^+^ cells were observed in TT. Investigation of the role of these cells in cancer pathology has revealed their heterogeneity. Meloni et al found a significant level of FOXP3 expression in CD8^+^CD57^+^ cells from lung cancer patients, which they attributed to the immunosuppressive component of the antitumor immune response [41, 47]. Although the increase in this lymphocyte subset in peripheral blood may contribute to impairments in cellular activation and may play a role in the decreased immunologic responsiveness observed in NSCLC patients, these cells are not able to penetrate the tumor. Our results suggest that CD8^+^CD57^+^ senescent cells are generated outside the tumor but do not enter it. However, the accumulation of CD8^+^CD39^+^ T cells in TT indicates a subpopulation of dysfunctional, exhausted CD8^+^ T cells, probably as a result of chronic stimulation.

The true role of CD20^+^ B cells in anticancer immunity remains controversial. There is considerable evidence of a tumor-protective function for B cells by the production of antitumor antibodies and the induction of cytotoxic immune responses, but other studies have suggested that B cells may also exert tumor-promoting functions [48]. It has recently been observed that B cells are paracrine mediators of solid tumor development cytokines such as interleukin IL-4, IL-10, and transforming growth factor β (TGFβ), which are among the most prominent immunosuppressive factors secreted by B cells in this setting [49–51]. In the present study, B-cells were practically non-existent in tumor nests and were preferentially located in the invasive margin when TT was analyzed by immunohistochemistry, while an inverse correlation between CD20^+^ B cells (high level in TT) and CD4^+^DR^+^ (activated T lymphocytes) was found in TT when analyzed by flow cytometry. In other words, an increase in B cells in TT was accompanied by a decrease in the percentage of CD4^+^DR^+^ cells and *vice-versa*. Furthermore, B cells in lung tumors can promote the suppression of CD8^+^ T-cell cytotoxicity and convert/recruit CD4^+^CD25^+^ FoxP3^+^ Tregs cells. In summary, our data suggest that the presence of B cell activities can also shift the balance of tumor specific immune response towards immunosuppression [48].

In the same way as effector T cells, NK cells are also involved in antitumor immunity. They comprise a heterogeneous functional population that can be divided into different subsets according to their surface expression [52, 53]. In the present NSCLC patients, a high percentage of NK cells with cytotoxic-phenotype (CD56^+^CD16^+^) were observed in peripheral blood, and these cells were also frequently located in DNTT. Around half of the tumor samples lacked HLA class I antigen expression (data not shown), and these two features make the cancer cells susceptible to NK cell attack. However, the most important study finding was the presence of two immune escape mechanisms: i) the exclusion of NK cells by cancer cells, given the very low percentage in ATT and the even lower percentage in TT; and ii) a progressive alteration in the phenotype of NK cells from HT to TT, leading to a non-cytotoxic phenotype or maturation alterations. Thus, whereas the dominant NK cell phenotype in the peripheral blood and DNTT was cytotoxic (CD56^+^ CD16^+^), the presence of cells with this phenotype was significantly decreased in ATT and even more so in TT. A low number of NK cells (CD56^+^ CD16^+^) in TT was previously attributed to their inefficient homing into malignant tissues [54, 55]. We also found a prevalence in TT of NK CD56^bright^ CD16^−^ (non-cytotoxic producing cytokines) and immature CD56^−^CD16^−^ (non-cytotoxic immature phenotype) subpopulations. These findings may have two hypothetical explanations: i) these NK phenotype changes may be induced directly at the tumor site, or there may be a preferential colonization of the tumor by NK cells at an early differentiation stage, following a specific chemokine-mediated migration pattern.

In summary, this study offers novel insights into the distribution and the phenotypic characteristics of different immune lymphocyte subpopulations. It provides the first comprehensive analysis of the immune infiltrate that shows quantitative and qualitative differences in subsets of immune infiltrate subpopulations among TT, ATT, and DNTT samples from the same patient. Our data showed how effector cells are mostly excluded from direct contact with cancer cells and are preferentially located in the stromal region, where an immunoprivileged space is generated, with a lack of cytotoxic response from T and NK cells, which may allow immune evasion in NSCLC patients and thereby contribute to cancer progression.

The immunologic response differed between adenocarcinomas and squamous cell carcinomas, with higher percentage of T lymphocytes detected by flow cytometry and immunohistochemistry in TT samples of the former, mainly located in the stroma. Immunohistochemistry studies showed that well-moderately differentiated tumors had a higher percentage of tumor infiltrating lymphocytes, mainly in invasive margins and tumor nests, and a higher percentage of macrophages in invasive margins. These findings may indicate an improved immunological response in well-moderately differentiated tumors, consistent with their better prognosis in comparison to poorly-differentiated tumors, which had a higher percentage of FoxP3^+^ T reg cells, indicating an immunosuppressive state.

These data suggest that the evasion of immune surveillance by tumors is favored by the lack of penetration of tumor nests by certain immune subpopulations. This is in line with previous findings and may reflect the difficulty that immune cells attracted to the tumor site have in overcoming physical and endothelial barriers and penetrating tumor nests.

The present data on most of the innate and adaptive immune cells infiltrating tumors provide a more complete picture of the immune reaction in NSCLC, further revealing the complex interactions of tumors with their microenvironment.

## MATERIALS AND METHODS

### Patients and Samples

The study included samples from 61 NSCLC patients under treatment at Virgen de las Nieves University Hospital (Granada, Spain). All surgical samples were obtained for therapeutic and diagnostic purposes. Demographic, clinical, and histological characteristics of the patients are exhibited in Table 1. Samples were obtained from 32 sex-matched healthy blood donors aged between 50 and 80 years (controls).

**Table 1:** Demographic, clinical, and histological characteristics of the study subjects

Variable	NSCLC *n* = 61	Healthy subjects *n* = 32
Gender *n* (%):		
Female	11 (18)	13 (40.6)
Male	50 (82)	19 (59.4)
Age,years, median (range)	66 (45–82)	62 (49–84)
Smoking history:		
Non-smoker	3 (5)	16 (50)
Ex-smoker	28 (46)	5 (15.6)
Smoker	30 (49)	15 (34.4)
Smoking pack-years, median (range)	55 (0–120)	7,78 (0–40)
Tumor size, median (range)	3.80 (0.5–9.5)	
Histological NSCLC subtype:		
Lung Adenocarcinoma	23 (36.1)	
Squamous cell carcinoma	32 (53,9)	
Metastasis from other cancer	6 (10)	
NSCLC stage:		
Stage IA	21 (36)	
Stage IB	18 (28)	
Stage IIA	13 (21.3)	
Stage IIB	7 (11.5)	
Stage IIIA	2 (3.3)	
T status:		
T1a	13 (20)	
T1b	10 (16)	
T2a	18 (29.5)	
T2b	11 (19)	
T3	8 (13.9)	
T4	1 (1.6)	
N status:		
Nx	4 (6.6)	
N0	45 (73.8)	
N1	6 (9.8)	
N2	6 (9.8)	
Differentiation:		
Moderate	27 (43)	
Good	11 (17.4)	
Poor	15 (24.6)	
SUVs PET, median (range)	9.52 (0–26.2)	

All blood samples were drawn after overnight fasting. Plasma samples obtained by centrifugation of part of each sample were stored at −20°C until use. The remaining blood samples were directly labeled with antibodies for flow cytometry (FCM).

Tumor samples were taken from primary malignant lung tumors of non-treated patients by excision of a fragment of tumor mass ≥ 100 mg during the initial surgery for the disease. After lung resection, half of the piece was immediately immersed in PBS solution and immediately analyzed by flow cytometry. The other half was fixed in buffered formalin and embedded in paraffin, and 3–4 micrometer sections were cut for conventional hematoxylin-eosin staining to evaluate tumor contents. All tumor samples had their paired adjacent tissue and distant non-tumor tissue samples. Only those with ≥ 50% tumor cells were selected (≈70% of samples had >70% tumor cells). Because preneoplastic molecular changes may take place in adjacent non-tumor tissue, distant non-tumor tissue samples (mass ≥ 500 mg) were taken from clinically tumor-free quadrants in the resected specimen as far as possible from the tumor. Adjacent tumor samples were taken from neighboring adjacent lung tissue with no macroscopic tumoral appearance located at approximately one centimeter from the periphery of the tumor. Distant non-tumor and adjacent tumor tissue samples underwent meticulous histological analysis to guarantee the complete absence of epithelial tumor cells.

Before the study, all medical records and tumor sections were reviewed by an oncologist and a surgical pathologist. Informed consent was obtained from all patients and healthy controls for participation in the study, which complied with the Helsinki Declaration of 1975, as revised in 1983, and was approved by the human research ethics committee of our institution.

Stages were determined after pathologic evaluation of resected specimens according to the 7th Edition of TNM in Lung Cancer of the International Association for the Study of Lung Cancer Staging Committee in 2009 [56].

### Flow cytometry analysis

Flow cytometry analyses were carried out in TT, ATT, and DNTT samples, which were washed twice with PBS and resuspended in 0.5 mL of PBS for FCM analysis. GentleMACS^TM^ Tubes (Miltenyi Biotec, Germany) were used for the dissociation and homogenization of samples. Each sample was processed twice, filtered, washed, and resuspended in 100 microliter of PBS; 20 microliter of sample tissue was added to labeled tubes containing specific antibodies for different lymphocytic populations and was incubated for 20 minutes at room temperature before addition of 1 ml red blood cell lysis buffer (BD Pharmalyse). Supplementary File 1 reports details of the flow cytometry analysis and the specific monoclonal antibodies used to identify the different lymphocytic populations.

### Immunohistochemistry and image analysis

Tissue samples were fixed in 10% neutral formalin and embedded in paraffin. Details of the immunohistochemistry staining and analysis are described in Supplementary File 1.

### Statistical analysis

All statistical analyses were performed using SPSS version 20.0 (IBM, Chicago IL). Shapiro Wilk and Kolmogorov-Smirnov tests were used to check the normality of variable distribution. Variables with normal distribution are expressed as means ± standard deviation and those with non-normal distribution as medians with interquartile range. Results were compared between groups using a *t-test* for normally-distributed variables and a non-parametric test (Wilcoxon or Mann Whitney Test) or ANOVA or the Kruskal Wallis test for those with non-normal distribution. Categorical variables (sex, tumor stage, tumor size) were grouped into two groups and analyzed using the chi-square (X^2^) or Fisher exact Test. Correlation coefficients were calculated to evaluate the strength of linear associations. *P* < 0.05 was considered statistically significant.

## SUPPLEMENTARY MATERIALS


