# Supplementary material for: Progressive changes in composition of lymphocytes in lung tissues from patients with non-small-cell lung cancer
Source: Oncotarget. 2016 Sep 26;7(44):71608–19. doi: 10.18632/oncotarget.12264 (PMC5342105; doi:10.18632/oncotarget.12264)
Supplement: Supplementary file 1 [file oncotarget-07-71608-s001.pdf]

Progressive changes in composition of lymphocytes in lung tissues from patients with non-small-cell lung cancer

Supplementary Materials

Supplementary File S1

Flow cytometry analysis

Specific monoclonal antibodies used to identify different lymphocyte populations were: anti- CD57-FITC, anti-CD4-PE, anti-CD8-Percp, anti CD8-APC H7, anti-CD45RO-Pecy7, anti HLA-DR-APC, anti-CD3-Pacific Blue (V450), Human regulatory T cell cocktail (anti-CD4-FITC/anti-CD25-Pecy7/anti-CD127-APC), anti-CD20-PE, anti-CD20-FITC, anti-CD20-Pacific Blue (V450), Simultest^{TM} (anti-CD3-FITC/CD56+CD16-PE), anti-CD183/PercpCy5 (CXCR3), anti-CD196/PE (CCR6), anti-CD56-PE, antiCD16-Percp, anti-CD45-Amcyam (V500), anti-EPCAM-Percp, anti-CD64-FITC, anti-CD11b-PE, anti-CD161-APC and anti-CD33-Percp (BD Bioscience, San José, California, USA). For multiparametric analysis, a minimum of 4,000 events in lung tissues or 30,000 in peripheral blood in the lymphocyte collection gate (CD45^{+} versus SSClow) were collected in a FACS CantoTM II analyzer. Data were analyzed using BD FACSDiva Software v.8.01 (BD).

An electronic gate was set for CD45+/SSC^{low} and a minimum of 10, 000 events in this gate were recorded for each sample. An additional electronic gate was made from the lymphocyte gate for T-cells (CD3^{+}) and either CD4^{+} or CD8^{+}, and the results were expressed as the relative percentage of each marker in the gate for CD4 or CD8 lymphocyte cells. CD4 (and CD8) cells can be classified as effector memory cells by expression of CD45RO. Human regulatory T cell cocktail was used to identify regulatory T cell subsets defined as CD127^{low} CD25^{bright} CD4^{+}. The amount of Treg was expressed as a percentage of total CD4 cells. Other T CD4 or CD8 subsets analyzed were CD39^{+} CD4^{+}, and CD39^{+} CD8^{+}, CD57^{+} CD8^{+} T cells. We also evaluated the presence of mature CD56 $^{+}$CD16^{+}, NK cells and subsets. NK cell subpopulations were determined by the selection of CD45+, CD3-, CD20- cells in the lymphocyte gate FSC^{low}/SSC^{low}. The gating strategy adopted offers the ability to detect several subpopulations within the plot: single positive CD16^{+}, single positive CD56^{+} and double positive CD16^{+} CD56^{+} mature NK-cells and low cytolytic CD56^{bright}, CD16$^{–}$ NK-cells. Immature NK-cells that express CD161 but not CD16 or CD56 could also be detected.

Similar to regulatory T cells, Th1 and Th17 were expressed as a percentage of total CD4^{+} T cells. Th1 was characterized as (CXCR3^{+}CCR6-CD4^{+}) cells and Th17 was characterized as (CXCR3-CCR6+CD4+) cells. Th1 Th17 data cells were only available from 14 patients. Finally, the presence of B- cells (CD20+) and granulocytes (CD11b+CD16+) was also investigated.

The percentage lymphocyte infiltration in each tumor was obtained by measuring CD45^{+}/SSC^{low} cells within the collection gate as a proportion of the total cell count. These data were used to analyze the relationship between the inflammatory infiltration from subsets and clinical- pathological features.

Immunohistochemistry and image analysis

Tissue samples were fixed in 10% neutral formalin and embedded in paraffin; 4 µm-thick sections were taken from the paraffin blocks, mounted on pretreated slices, and stained for CD45, CD20, CD3, CD4 CD8 or CD68., using prediluted antibodies supplied by Master Diagnóstic (CD45: Ref MAD-002066QD, batch:0108-01; CD20: Ref MAD-002037QD, batch 0267-10, clone L26; CD3: Ref MAD00621QD, batch: 0117-02, clone EP41; CD4: Ref: MAD-00600QD, batch:0107-02-10, clone EP 204; CD8: Ref MAD-000618QD, batch 0183-11, clone SP16; CD68: Ref MAD-002097QD, batch 0175-07). Immunohistochemical staining was carried out using the of UltraVision Quanto (peroxidase) immunohistochemistry detection system (MAD-021881QK/MAD-001881QK Master Diagnóstica) following manufacturer's instructions. In short, paraffin sections were dewaxed and endogenous peroxidase activity blocked in a solution of hydrogen peroxide for 10 min. For antigen retrieval, sections were treated with sodium citrate buffer (pH 6.0). After incubation with the primary antibodies for 16 hours at 4ºC and washing 3 times with PBS for 5 minutes, tissue sections were again incubated for 10 min with the primary antibodies Amplificator (UltraVisionQuanto) and then washed, and incubated with the polymer Quanto (Ultra Vision Quanto) for 10 minutes. DAB was used as chromogen and sections were counterstained with hematoxylin.

No staining was obtained when non-immune serum or PBS was used instead of the primary antibody, confirming the specificity of the latter. All specimens were

primary tumors with no previous treatment that may affect the immunohistochemical results.

Lymphocyte infiltration was evaluated into three groups based on their localization: (a) those infiltrated within cancer cell nests, (b) those distributed in the cancer stroma, and (c) those present along the invasive margin (tumor-host interface).

The number of cells stained by each antibody was semi-quantitatively evaluated using the following scores: 1+ (minimal - stained cells practically absent, < 5 stained cells per high power field (HPF) 40×), 2+ (mild infiltrate, 6–25 stained cells per HPF 40×), 3+ (moderate, 26−50 stained cells per HPF 40×), 4+ (severe – intense, > 50 stained cells per HPF 40×).

Manual evaluation of stained immune cells was conducted in duplicate by two independent observers blinded to the clinicopathological findings. These data were used in the statistical analysis. Figure 3 depicts representative examples of immunohistochemical findings in intraepithelial tumor-infiltrating lymphocytes, showing stromal and invasive margin patterns.
